# Bioelectrical Impedance Analysis Can Be an Effective Tool for Screening Fatty Liver in Patients with Suspected Liver Disease

**DOI:** 10.3390/healthcare10112268

**Published:** 2022-11-11

**Authors:** Jin Wook Choi, Jeong-Ju Yoo, Sang Gyune Kim, Young Seok Kim

**Affiliations:** Division of Gastroenterology and Hepatology, Department of Internal Medicine, Soonchunhyang University Bucheon Hospital, 170 Jomaru-ro, Bucheon 14584, Korea

**Keywords:** bioelectrical impedance analysis, controlled attenuation parameter, fatty liver, transient elastography

## Abstract

Background and study aims: Although abdominal ultrasound (USG) or controlled attenuation parameter (CAP) score of transient elastography (TE) is recommended for the diagnosis of fatty liver, issues regarding cost and accessibility still exist. The aim of this study was to evaluate if bioelectrical impedance analysis (BIA) can be used as a reliable screening tool for fatty liver. Patients and methods: A total of 249 patients who underwent all three tests including TE, BIA, and USG were enrolled. The correlation between fat mass measured by BIA, CAP score of TE, and fatty liver grade measured by USG was analyzed. In addition, the cut-off value of BIA which can predict the fatty liver grade was calculated. Results: Fat mass index (FMI) assessed by BIA increased significantly along with the rise in fatty liver grade measured by USG (normal: 6.2 ± 2.4, Gr I: 8.0 ± 3.7, Gr II: 10.6 ± 3.5, Gr III: 10.7 ± 3.7 kg/m^2^, *p* < 0.001). In addition, a positive correlation was found between the CAP score of TE and the FMI of BIA. Additionally, a total body fat mass increase by 24.3% or 29.8% in men and 34.8% or 35.1% in women increased the possibility of developing any grade of fatty liver or significant fatty liver (≥Gr II fatty liver), respectively. Conclusion: The total fat or fat mass index of BIA was related to fatty liver as assessed by ultrasound or CAP score, and area under the receiver operating characteristic (AUROC) was about 0.8. Thus, BIA can be used as a screening tool for fatty liver in patients with suspected liver disease.

## 1. Introduction

Nonalcoholic fatty liver disease (NAFLD) is the leading cause of liver transplantation and hepatocellular carcinoma [1,2]. Recently, the continuous increase of fatty liver prevalence and its sequela has become a global problem. The estimated prevalence of NAFLD has reached 25.2% worldwide and 30% in the United States [3,4,5]. NAFLD is strongly associated with obesity and metabolic syndrome, and thus early diagnosis of fatty liver and lifestyle corrections in accordance with treatment measures have become important health issues [6,7].

In the past, there was relatively low clinical interest in steatosis compared to fibrosis in the field of fatty liver studies. In fact, many studies reported that nonalcoholic steatohepatitis (NASH) or steatosis other than fibrosis had no significant relationship with the prognosis [8,9]. However, in a recent large cohort study based on histological findings, not only fibrosis or NASH, but even simple steatosis increased the overall mortality compared to the control group [10]. In particular, steatosis itself increased cancer-related mortality, cardiovascular disease mortality, and cirrhosis-related mortality [11,12]. This clinical significance of steatosis is not limited to NAFLD. It has been reported that when fatty liver is accompanied by chronic hepatitis B or chronic hepatitis C, it can affect the overall prognosis or treatment response of the patients [13,14]. Based on this recognition, the concept of metabolic associated fatty liver disease (MAFLD) that can include a wider range of fatty liver patients has recently emerged [15]. Thus, suspicion and early diagnosis of steatosis has gained more clinical importance.

Abdominal ultrasound (USG) or the recently introduced controlled attenuation parameter (CAP) score of transient elastography (TE) can be used to diagnose fatty liver [16]. USG is recommended as a screening method for fatty liver in most clinical guidelines [17,18]. CAP of TE is also widely used for the diagnosis of fatty liver. However, existing methods for diagnosing fatty liver have some limitations. First, USG and TE are relatively expensive for repeated testing or use in general practice [19,20]. The average cost for conducting USG is USD 420 and for TE, USD 240 [19]. Second, both test methods can be performed only in specialized medical institutions.

Bioelectrical impedance analysis (BIA) is a widely used, non-invasive method to estimate body composition with high accuracy and reproducibility [21]. BIA sends a weak electrical current through the body and calculates the impedance to measure intracellular water, muscle mass, and body fat [22]. Advantages of BIA include quick (less than 5 min) and simple usage, no requirements for a professional assistant, and easy, repeated measures. Moreover, BIA is equipped in most health centers in Korea, and thus the general public can have easy access to this method.

Despite these advantages, BIA has never been studied in relation to the diagnosis or screening of fatty liver. Fatty liver is more likely to occur as total body fat increases. Therefore, we assumed that fatty liver could be predicted using factors such as total body fat that can be measured in BIA. The aim of this study was to evaluate BIA as a screening method for fatty liver, in comparison to USG or CAP score measured by TE.

## 2. Patients and Methods

### 2.1. Patients and Study Design

This study was a single-center, retrospective study and included patients who underwent BIA tests between 16 July 2018 and 27 May 2020 at a tertiary referral hospital. The inclusion criteria were: (a) patients who underwent all three tests including USG, TE and BIA for any type of liver disease, and (b) aged more than 19 years. Exclusion criteria were: (a) patients without laboratory data, and (b) aged less than 19 years. Accordingly, a total of 303 patients were examined with USG, TE and BIA, and 54 patients without laboratory data were excluded. As a result, 249 patients that underwent all three tests of USG, TE, and BIA were selected for final analysis.

The protocol for the study was approved by the Institutional Review Board of Soonchunhyang University Bucheon Hospital (SCHBC-2021-06-030). This study conformed to the ethical guidelines of the World Medical Association Declaration of Helsinki. The requirement for informed consent from individual subjects was waived due to the retrospective nature of the study.

### 2.2. USG

USG was conducted using the LOGIQ (E9 model; GE Healthcare, Chicago, IL, USA). Fatty liver grades were classified as normal, mild, moderate, and severe according to the following classification criteria [23]: (a) normal grade for when the echotexture of the liver shows no abnormal findings; (b) mild grade for a slight and diffuse increase in liver echogenicity with normal visualization of the diaphragm and portal vein wall; (c) moderate grade for a moderate increase of liver echogenicity with slightly impaired appearance of the portal vein wall and diaphragm; and (d) severe grade for the marked increase of liver echogenicity with poor or no visualization of the portal vein wall, diaphragm, and posterior part of the right liver lobe. Abdominal USG and fatty liver grading were performed by three experts with more than ten years of experience.

### 2.3. TE

TE was conducted using the Fibroscan^®^ (E360 model; Echosens, Paris, France). Hepatic steatosis score (dB/m) known as the CAP score and hepatic fibrosis score (kPa) was measured by Fibroscan^®^. Measurements were repeated at least ten times and the median value was recorded. Ten measurements were performed with a success rate of at least 60%. Only procedures with at least ten valid measurements with interquartile range/median value <0.3 were considered valid [24]. TE was conducted by expert physicians with extensive experience of TE in more than 1000 cases.

### 2.4. BIA

BIA was conducted using the Inbody^®^ (970 model; Inbody Co., Ltd., Seoul, Korea). Patients were restricted from water, caffeine, food, and exercise for 4 h before the BIA test [25]. The BIA test was performed solely by the patient without the help of an assistant. Body weight, body muscle mass, body muscle percentage, body fat mass, body fat percentage, total body water, basic metabolic rate and waist-to-hip ratio were measured.

### 2.5. Data Collection and Endpoints

For patients enrolled in the study, multiple factors were collected including age, sex, height, weight, etiology of liver disease (viral hepatitis vs. non-viral hepatitis), blood test values such as white blood cell, C-reactive protein, hemoglobin, platelet, aspartate transaminase, alanine transaminase, total bilirubin, albumin, serum sodium, serum creatinine, total cholesterol, high density lipoprotein, low density lipoprotein, triglyceride, prothrombin time-international normalized ratio (PT-INR), serum glucose, glycated hemoglobin (HbA1c) and serum insulin.

Based on such laboratory tests, Fibrosis-4 (FIB-4) score, AST to Platelet Ratio Index score (APRI), and NAFLD fibrosis score were measured as supportive indicators for liver fibrosis. In addition, Homeostatic Model Assessment of Insulin Resistance (HOMA-IR) was calculated. Fat mass index (FMI) was defined as a value obtained by dividing BIA’s total body fat mass (kg) by the square of the height [26]. As a subgroup study, patients who had abdominal computed tomography (CT) three months before or after BIA were analyzed for visceral fat and muscle mass using axial images at the L3 spine level [27,28].

The primary endpoint of this study was to evaluate the presence of a statistically significant relationship between fatty liver grade assessed by USG, CAP score measured by TE and body fat mass estimated by BIA. As a secondary endpoint, the cut-off value of BIA to predict fatty liver grade was calculated.

### 2.6. Statistical Analysis

Frequencies and percentages were used for the descriptive statistics. Significant differences between the groups were investigated using the chi-squared test for categorical variables and Student’s t-test for continuous variables. Linear regression analysis was applied to evaluate the relationships between steatosis and other factors. As it was judged that the collinearity of the items was not large, a linear regression analysis method was selected rather than principal components analysis [29,30]. Instead, for items with known collinearity (e.g., body mass index, height), only one clinically important item among the two was included as a multivariate variable. Receiver operating characteristic analysis was employed to determine the sensitivity and specificity in detecting fatty liver. All statistical analyses were performed using R (version 4.1.0; The R Foundation for Statistical Computing, Vienna, Austria). Statistical significance was set at *p* < 0.05.

## 3. Results

### 3.1. Baseline Characteristics

The characteristics of the patients are described in Table 1. The most common etiology of liver diseases was NAFLD accounting for 73.9%, followed by alcoholic hepatitis (16.9%), chronic hepatitis B (8.0%), and chronic hepatitis C (1.2%). Mean body mass index (BMI) was 26.3 ± 5.2 kg/m^2^, and 26.5% of the patients had diabetes.

As for the severity of fatty liver measured by USG, 31.0% of the patients were of normal grade, 25.7% mild grade, 22.4% moderate, and 20.8% severe. CAP score measured by TE showed a mean value of 264.3 ± 59.9 dB. Regarding the average values of body measurement by BIA, the absolute body fat mass and percent body fat mass corresponded to 23.2 ± 10.6 kg and 31.5 ± 8.7%, respectively.

### 3.2. BIA and Fatty Liver Grade on USG

Table 2 displays the baseline characteristics according to the severity of fatty liver measured by USG. Serum levels of total cholesterol (*p* < 0.001), LDL (*p* < 0.05), and TG (*p* < 0.05) tended to increase as the severity of fatty liver assessed by USG increased. As the fatty liver grade assessed by USG grew higher, the fat mass index in BIA increased significantly (normal: 6.2 ± 2.4, Gr I: 8.0 ± 3.7, Gr II: 10.6 ± 3.5, Gr III: 10.7 ± 3.7 kg/m^2^, *p* < 0.001) and the percent body fat mass of BIA also increased (normal: 26.8 ± 7.9, Gr I: 30.6 ± 8.8, Gr II: 35.9 ± 7.2, Gr III: 34.8 ± 7.5 %, *p* < 0.001). BMI values also escalated as the severity of fatty liver increased (*p* < 0.001). The visceral fat mass measured at L3 level by abdominal CT also showed a tendency to expand in proportion to the severity of fatty liver (*p* < 0.001).

### 3.3. BIA and CAP Score of TE

The results of linear regression analysis regarding the CAP score are displayed in Table 3. The CAP score showed positive correlations with serum total cholesterol, LDL, and TG (*p* < 0.001). Fatty liver severity on USG had a positive correlation with CAP score values (Gr I: regression coefficient [β] 37.88, Gr II: β 90.68, Gr III: β 110.35, *p* < 0.001). CAP score of TE showed a positive correlation with the absolute body fat mass, percent body fat mass, and fat mass index in BIA (absolute body fat mass, β 3.24, 95% confidence interval [CI] 2.66–3.83, *p* < 0.001; percent body fat mass, β 3.16, 95% CI 2.40–3.93, *p* < 0.001; FMI, β 8.26, 95% CI 6.58–9.95, *p* < 0.001). Multivariable analysis showed a statistically significant, positive correlation with the CAP score in cases of any grades of fatty liver in USG (*p* < 0.001). Other significant factors in multivariable analysis were skeletal muscle, albumin, and c-reactive protein (supplementary material Figure S1).

### 3.4. Accuracy of BIA Predicting Fatty Liver Evaluated by USG or CAP Score

**Figure 1:**
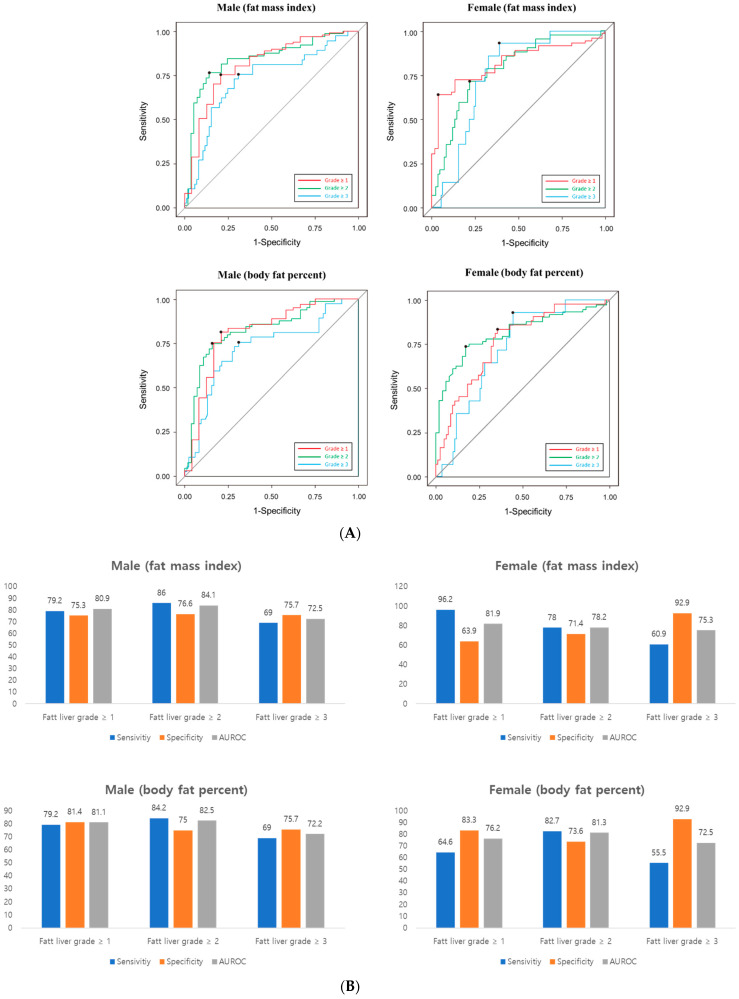
(**A**) Receiver operating characteristic (ROC) analysis (**B**) Accuracy prediction of fatty grade using BIA’s FMI and body fat percent. Left. Male, right. female. AUROC, area under the ROC.

Next, we calculated the cut-off value of FMI or body fat percent that can predict the grade of fatty liver on USG (Figure 1A). This cut-off was presented differently by sex.

The cut-off value of FMI in men was as follows: any grade of fatty liver, 6.46 kg/m^2^; fatty liver grade ≥ II, 7.89 kg/m^2^; fatty liver grade ≥ III, 8.26 kg/m^2^. In female, the cut-off value was as follows: any grade of fatty liver, 9.70 kg/m^2^; fatty liver grade ≥ II, 9.70 kg/m^2^; fatty liver grade ≥ III, 9.75 kg/m^2^. The cut-off value of body fat percent in men was as follows: any grade of fatty liver, 24.35%; fatty liver grade ≥ II, 29.82%; fatty liver grade ≥ III, 30.43%. In female, the cut-off value was as follows: any grade of fatty liver, 34.80%; fatty liver grade ≥ II, 35.17%; fatty liver grade ≥ III, 36.16%. AUROC graphs for each cut-off are presented in the supplementary materials. The sensitivity of fatty liver grade using BIA was 79% in male and 64–96% in female. The specificity was 75–80% in male and 63–92% in female (Figure 1B). AUC was approximately 72–80% for both men and women (Figure 1A).

**Figure 2:**
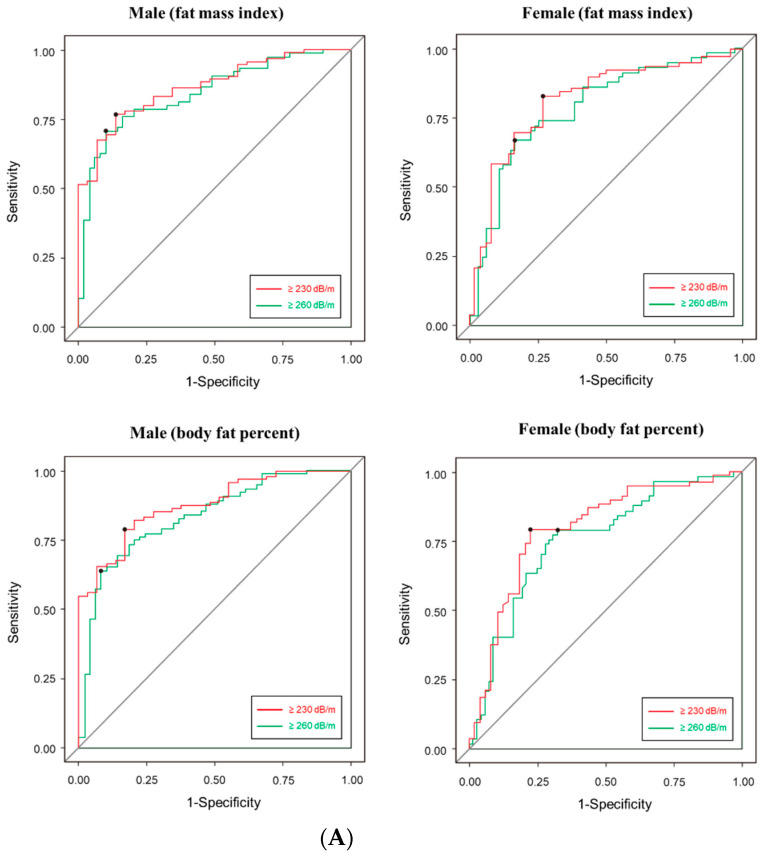

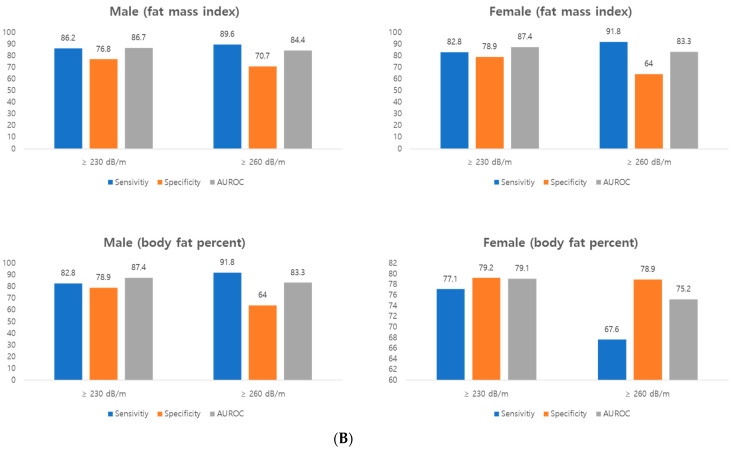
(**A**) Receiver operating characteristic (ROC) analysis (**B**) Accuracy prediction of controlled attenuation parameter score of transient elastography using BIA’s FMI and body fat percent. Left. Male, right. female. AUROC, area under the ROC.

Finally, we calculated the cut-off value of body fat mass that can predict the CAP score of TE (Figure 2A). The cut-off value of FMI in men was as follows: CAP ≥ 230 dB/m, 6.49 kg/m^2^; CAP ≥ 260 dB/m, 7.89 kg/m^2^. In female, the cut-off value was as follows: CAP ≥ 230 dB/m, 7.15 kg/m^2^; CAP ≥ 260 dB/m, 9.70 kg/m^2^. The cut-off value of body fat percent in men was as follows: CAP ≥ 230 dB/m, 24.35%; CAP ≥ 260 dB/m, 29.82%. In female, the cut-off value was as follows: CAP ≥ 230 dB/m, 34.80%; CAP ≥ 260 dB/m. AUROC graphs for each cut-off are presented in the supplementary materials. The sensitivity of CAP score using BIA was slightly higher in male (82–91%) than female (67–82%). The specificity was similar between male (64–78%) and female (64–92%) (Figure 2B). AUC was slightly was higher in male (83–87%) than female (75–87%) (Figure 2A).

## 4. Discussion

This study found that BIA was highly correlated with USG or the CAP score of TE. In addition, a total body fat mass increase of 25% or 31% in men and 32% or 34% in women raises the possibility of developing any grade of fatty liver or significant fatty liver (≥Gr II fatty liver), respectively. To our knowledge, this is the first study to determine the associations between BIA and USG, and BIA and TE.

According to several guidelines, USG is primarily recommended as a non-invasive test for diagnosing steatosis. However, the interpretation of USG is subjective and it is difficult to conduct USG on obese subjects. Additionally, issues of low sensitivity exist for livers with less than 30% fat. In addition to this imaging test, steatosis can be diagnosed using methods such as fatty liver index, NAFLD liver fat score, or hepatic steatosis index. However, these serological methods have disadvantages such as low accuracy and blood test requirements. Therefore, rather than accurately diagnosing steatosis in most cases, serological markers can help physicians decide whether further assessments are required for patients with suspected hepatic steatosis [16]. In this regard, BIA has several advantages compared to serologic markers, as BIA does not require blood tests and it can be repeatedly measured by the patient.

Another finding of our study was the accuracy of BIA for predicting fatty liver. Although there were some differences in sensitivity and specificity according to fatty liver grade, the ability to diagnose fatty liver with BIA was high with AUC 0.8 or higher in both males and females. The rationale for using BIA for the purpose of screening fatty liver is that BIA has a high correlation with steatosis of liver histology. There were two studies that reported a high degree of correlation between BIA and histologic steatosis from a living donor of liver transplantation [31,32]. In particular, BIA was highly correlated with macrovesicular steatosis [31]. High levels of total body fat can eventually lead to excessive liver adiposity, increasing the possibility of fatty liver [33,34].

Although there are not many existing studies yet, there have been previous studies showing that BIA is correlated with the existing fatty liver test method such as TE [35]. According to this study, the cut-off value of total body fat in BIA for suspected fatty liver was 24% for men and 34% for women. In Iran, the cut-off of BIA was suggested for the CAP score of TE, and similar to our study, the results were 29% for men and 35% for women. This suggests that the cut-off of total body fat for fatty liver may be similar among different races. In addition, the literature comparing the cut-off of BIA and NAFLD diagnosis through USG in pediatric Taiwanese was also referred to in the study. In the case of male patients, the AUC reached 0.854, which showed the possibility of expanding the research as a screening tool through BIA in the future to all age groups, not adults [36]. At present, no previous studies have suggested a BIA cut-off value based on USG, and thus further study is necessary.

In order to compare this study with the study conducted on actual living patients, more prior research for reference were additionally investigated. However, there were only literatures on the relationship between BIA value and liver steatosis in the liver resected for hepatic transplantation. After the start of this study, literatures that attempted to relate fatty liver with BIA on children or other races began to be published, but there was a disadvantage that the number of patients was small and the statistical significance was not high. However, in this study, we succeeded in securing statistical significance through a sufficient group of patients, and also conducted a study using all USG, TE, and BIA simultaneously for the study group. Therefore, it is expected that it can be used as a precedent study for future studies.

Although we presented BIA as a screening tool that can be easily accessed by the general public, BIA is still a tool with many limitations. First, BIA does not directly measure fat or muscles, but uses an electric current for indirect assessments, and thus an error value exists depending on the water status. Second, several companies manufacture and sell BIA, but these companies have yet to provide validation. Additionally, some models cannot separately measure visceral fat, but can only assess total body fat. Third, its use may be restricted in patients with advanced liver diseases such as significant fibrosis or ascites. Lastly, BIA shows adequate predictive power in most cross-sectional studies, and further studies are needed for its usefulness in evaluating disease progression or response to treatment.

Our study also has several limitations. First, our study included various etiologies whereas other studies analyzed BIA results limited to NAFLD. However, when considering the recent emerging concept of MAFLD, we judged it to be clinically meaningful as the presence of fatty liver can affect the prognosis regardless of viral infection or alcohol. Second, our proposed cut-off value is limited to Asians, and thus cannot be applied to other races. However, as introduced earlier, the Iranian case reported similar results to our cut-off values, so validation is required in other races as well. Finally, our BIA model does not accurately distinguish the degree of visceral fat, and thus we lacked additional information of visceral fat other than total body fat.

## 5. Conclusions

BIA can be purposed as a screening test for steatosis in patients with suspected liver disease including both men and women. Further studies are needed to determine whether BIA is more useful than other anthropometric parameters or serological panels.

## Figures and Tables

**Table 1:** Baseline characteristics.

Variable	Total (*n* = 249)	Male (*n* = 124)	Female (*n* = 125)
Age	45.5 ± 13.3	42.5 ± 12.5	48.5 ± 13.5
Etiology			
Viral	23 (9.2%)	12 (9.7%)	11 (8.8%)
Non-viral	226 (90.8%)	112 (90.3%)	114 (91.2%)
Etiology			
Hepatitis B virus	20 (8.0%)	11 (8.9%)	9 (7.2%)
Hepatitis C virus	3 (1.2%)	1 (0.8%)	2 (1.6%)
Alcohol	42 (16.9%)	29 (23.4%)	13 (10.4%)
Etc.	184 (73.9%)	83 (66.9%)	101 (80.8%)
White blood cell (/μL)	6664.0 ± 3031.8	7403.7 ± 3497.8	5948.1 ± 2296.0
C-reactive protein (mg/dL)	0.8 ± 2.2	0.8 ± 2.3	0.8 ± 2.0
Hemoglobin (g/dL)	13.6 ± 2.3	14.7 ± 2.4	12.6 ± 1.6
Platelet (1 k/μL)	212.4 ± 78.6	219.8 ± 75.5	205.3 ± 81.2
Aspartate aminotransferase (U/L)	53 [33, 126]	53 [33, 126]	53 [33, 129]
Alanine aminotransferase (U/L)	66 [36, 120]	67 [36, 124]	66 [36, 121]
Total bilirubin (mg/dL)	1.3 ± 2.0	1.6 ± 2.5	1.1 ± 1.2
Albumin (g/dL)	4.3 ± 0.6	4.5 ± 0.6	4.2 ± 0.6
Serum sodium (mmol/L)	140.5 ± 3.1	140.6 ± 3.0	140.4 ± 3.1
Creatinine (mg/dL)	1.0 ± 0.4	1.1 ± 0.3	0.9 ± 0.5
Total cholesterol (mg/dL)	186.9 ± 50.2	189.8 ± 49.3	184.0 ± 51.2
High density lipoprotein (mg/dL)	45.1 ± 17.6	41.4 ± 13.8	48.8 ± 20.1
Low density lipoprotein (mg/dL)	115.6 ± 39.4	118.5 ± 40.6	112.5 ± 38.0
Triglyceride (mg/dL)	173.9 ± 156.9	213.7 ± 194.1	134.1 ± 92.4
PT-INR	1.0 ± 0.3	1.0 ± 0.2	1.1 ± 0.5
Glucose (mg/dL)	122.8 ± 54.8	121.6 ± 53.4	124.1 ± 56.3
Diabetes			
No	183 (73.5%)	90 (72.6%)	93 (74.4%)
Yes	66 (26.5%)	34 (27.4%)	32 (25.6%)
HbA1c (%)	5.9 ± 0.9	5.8 ± 0.9	6.1 ± 0.8
Insulin (uIU/mL)	18.4 ± 14.7	18.8 ± 15.3	18.0 ± 14.1
Fibroscan^®^ stiffness	6.6 [5.3, 11.5],	6.6 [5.3, 11.5],	6.6 [5.3, 11.6]
Fibroscan^®^ steatosis (CAP score)	264.3 ± 59.9	276.0 ± 62.6	252.7 ± 55.0
Sonographic fatty liver grade			
Normal	76 (31.0%)	24 (19.8%)	52 (41.9%)
Mild	63 (25.7%)	33 (27.3%)	30 (24.2%)
Moderate	55 (22.4%)	27 (22.3%)	28 (22.6%)
Severe	51 (20.8%)	37 (30.6%)	14 (11.3%)
Body weight (kg)	71.5 ± 17.8	80.2 ± 17.7	62.8 ± 13.1
Skeletal muscle mass (kg)	26.6 ± 6.3	31.4 ± 5.0	21.9 ± 3.2
Skeletal muscle mass (%)	37.6 ± 4.8	39.8 ± 4.3	35.5 ± 4.2
Muscle mass (cm^2^)	121.2 ± 32.4	144.2 ± 33.1	104.1 ± 18.2
Body fat mass (kg)	23.2 ± 10.6	24.2 ± 11.5	22.3 ± 9.5
Body fat mass (%)	31.5 ± 8.7	28.8 ± 8.3	34.2 ± 8.3
Visceral fat (cm^2^)	120.5 ± 70.0	143.1 ± 76.3	103.4 ± 59.9
Fat mass index (kg/m^2^)	8.6 ± 3.8	8.3 ± 3.8	9.0 ± 3.7
Total body water (L)	35.4 ± 7.6	41.1 ± 6.0	29.8 ± 3.9
Base metabolic rate (kcal)	1410.9 ± 223.9	1578.3 ± 177.2	1244.8 ± 115.2
Waist to hip ratio	0.9 ± 0.1	0.9 ± 0.1	0.9 ± 0.1
Body mass index	26.3 ± 5.2	27.3 ± 5.4	25.3 ± 4.8
FIB-4 score	4.4 ± 7.7	4.3 ± 8.9	4.6 ± 6.4
APRI score	2.7 ± 7.8	2.5 ± 7.4	2.8 ± 8.3
NAFLD fibrosis score	−1.6 ± 2.2	−1.9 ± 2.2	−1.2 ± 2.2
HOMA-IR	5.4 ± 4.8	5.4 ± 4.3	5.4 ± 5.2

Data were reported as mean ± standard deviation or median [interquartile range] for continuous variables and proportion (%) for categorical variables. PT-INR, prothrombin time-international normalized ratio; HbA1c, glycated hemoglobin; CAP, controlled attenuation parameter; FIB-4, Fibrosis-4; APRI, aspartate transaminase to Platelet Ratio Index; NAFLD, nonalcoholic fatty liver disease; HOMA-IR, Homeostatic Model Assessment of Insulin Resistance.

**Table 2:** Baseline characteristics according to fatty liver grade of sonography.

Variable	Total	Normal	Mild	Moderate	Severe	*p*
	(*n* = 245)	(*n* = 76)	(*n* = 63)	(*n* = 55)	(*n* = 51)	
Age	45.6 ± 13.4	49.4 ± 13.8	49.4 ± 11.6	39.9 ± 13.0	41.5 ± 12.0	<0.001
Sex						<0.001
Male	121 (49.4%)	24 (31.6%)	33 (52.4%)	27 (49.1%)	37 (72.5%)	
Female	124 (50.6%)	52 (68.4%)	30 (47.6%)	28 (50.9%)	14 (27.5%)	
Etiology						<0.001
Viral	23 (9.4%)	17 (22.4%)	4 (6.3%)	1 (1.8%)	1 (2.0%)	
Non-viral	222 (90.6%)	59 (77.6%)	59 (93.7%)	54 (98.2%)	50 (98.0%)	
Etiology						-
Hepatitis B virus	20 (8.2%)	15 (19.7%)	4 (6.3%)	1 (1.8%)	0 (0.0%)	
Hepatitis C virus	3 (1.2%)	2 (2.6%)	0 (0.0%)	0 (0.0%)	1 (2.0%)	
Alcohol	41 (16.7%)	13 (17.1%)	15 (23.8%)	7 (12.7%)	6 (11.8%)	
Etc.	181 (73.9%)	46 (60.5%)	44 (69.8%)	47 (85.5%)	44 (86.3%)	
White blood cell (/μL)	6661.7 ± 3044.0	6484.6 ± 4245.0	6112.9 ± 1898.4	6966.3 ± 2572.0	7282.4 ± 2328.9	0.180
C-reactive protein (mg/dL)	0.8 ± 2.2	1.0 ± 2.4	0.7 ± 1.9	0.5 ± 1.1	0.8 ± 2.9	0.774
Hemoglobin (g/dL)	13.6 ± 2.3	12.4 ± 2.1	13.7 ± 1.8	14.3 ± 1.8	14.6 ± 2.7	<0.001
Platelet (1 k/μL)	212.0 ± 78.8	185.6 ± 80.2	207.7 ± 77.3	240.1 ± 73.0	227.1 ± 72.7	<0.001
Aspartate aminotransferase (U/L)	53 [33, 129]	53 [33, 126.8]	52.5 [33, 126]	53 [33, 126.8]	48 [32, 115]	0.226
Alanine aminotransferase (U/L)	66 [36, 121]	66.5 [36, 121.8]	67 [36.25, 123.3]	66 [36.75, 120.3]	65 [35, 120]	0.084
Total bilirubin (mg/dL)	1.4 ± 2.0	1.6 ± 3.0	1.5 ± 1.8	1.2 ± 1.2	1.0 ± 0.7	0.269
Albumin (g/dL)	4.3 ± 0.6	4.0 ± 0.6	4.3 ± 0.5	4.6 ± 0.6	4.7 ± 0.5	<0.001
Serum sodium (mmol/L)	140.4 ± 3.1	139.5 ± 3.5	140.6 ± 2.8	141.3 ± 2.6	140.7 ± 2.8	0.006
Creatinine (mg/dL)	1.0 ± 0.4	1.0 ± 0.7	0.9 ± 0.2	0.9 ± 0.2	1.0 ± 0.3	0.493
Total cholesterol (mg/dL)	186.6 ± 50.2	166.5 ± 40.8	189.0 ± 47.8	199.2 ± 56.2	199.7 ± 50.8	<0.001
High density lipoprotein (mg/dL)	45.1 ± 17.7	45.0 ± 19.0	44.3 ± 18.5	47.0 ± 18.8	44.2 ± 13.1	0.830
Low density lipoprotein (mg/dL)	114.7 ± 39.1	100.4 ± 26.5	110.6 ± 42.0	119.1 ± 40.9	125.9 ± 39.6	0.016
Triglyceride (mg/dL)	174.0 ± 158.0	111.7 ± 62.4	193.5 ± 188.2	204.2 ± 196.8	209.0 ± 146.0	0.001
PT-INR	1.0 ± 0.4	1.1 ± 0.2	1.0 ± 0.2	1.0 ± 0.1	1.1 ± 0.7	0.294
Glucose (mg/dL)	123.2 ± 55.2	131.5 ± 77.4	120.8 ± 41.2	112.9 ± 25.8	125.0 ± 53.1	0.280
Diabetes						0.357
No	180 (73.5%)	60 (78.9%)	47 (74.6%)	40 (72.7%)	33 (64.7%)	
Yes	65 (26.5%)	16 (21.1%)	16 (25.4%)	15 (27.3%)	18 (35.3%)	
HbA1c (%)	5.9 ± 0.9	6.3 ± 1.7	6.1 ± 0.8	5.8 ± 0.7	5.8 ± 0.7	0.238
Insulin (uIU/mL)	18.5 ± 14.7	15.6 ± 13.3	18.4 ± 16.7	19.7 ± 14.0	21.2 ± 14.7	0.193
Fibroscan^®^ stiffness	6.6 [5.3, 11.6]	6.6 [5.3, 11.525]	6.6 [5.3, 11.5]	6.65 [5.3, 11.525]	6.5 [5.3, 11]	0.002
Fibroscan^®^ steatosis	263.0 ± 59.5	210.0 ± 39.0	247.9 ± 38.5	300.7 ± 37.0	320.3 ± 47.0	<0.001
Body weight (kg)	71.3 ± 17.9	60.1 ± 11.5	67.9 ± 14.9	79.0 ± 17.7	84.0 ± 17.8	<0.001
Skeletal muscle mass (kg)	26.6 ± 6.3	23.8 ± 5.2	25.7 ± 5.8	27.9 ± 6.5	30.3 ± 6.3	<0.001
Skeletal muscle mass (%)	37.6 ± 4.7	39.5 ± 4.4	38.2 ± 5.1	35.4 ± 4.1	36.3 ± 4.1	<0.001
Body fat mass (kg)	23.2 ± 10.6	16.2 ± 6.7	21.3 ± 9.3	28.8 ± 10.1	29.9 ± 10.3	<0.001
Body fat mass (%)	31.5 ± 8.7	26.8 ± 7.9	30.6 ± 8.8	35.9 ± 7.2	34.8 ± 7.5	<0.001
Fat mass index (kg/m^2^)	8.6 ± 3.8	6.2 ± 2.4	8.0 ± 3.7	10.6 ± 3.5	10.7 ± 3.7	<0.001
Total body water (L)	35.3 ± 7.6	32.1 ± 6.4	34.2 ± 6.9	36.9 ± 7.9	39.9 ± 7.6	<0.001
Base metabolic rate (kcal)	1408.6 ± 224.6	1314.4 ± 186.5	1375.7 ± 203.2	1454.2 ± 232.6	1540.5 ± 222.7	<0.001
Waist to hip ratio	0.9 ± 0.1	0.9 ± 0.1	0.9 ± 0.1	1.0 ± 0.1	1.0 ± 0.1	<0.001
Body mass index	26.2 ± 5.2	22.8 ± 3.3	25.4 ± 4.7	28.9 ± 4.8	29.5 ± 5.0	<0.001
FIB-4 score	4.5 ± 7.8	5.5 ± 6.7	4.4 ± 6.2	3.4 ± 6.7	4.2 ± 11.4	0.501
APRI score	2.7 ± 7.9	2.8 ± 5.1	4.1 ± 12.6	2.5 ± 7.5	1.0 ± 1.7	0.226
NAFLD fibrosis score	−1.5 ± 2.2	−0.9 ± 2.5	−1.5 ± 1.9	−2.2 ± 2.1	−1.9 ± 2.2	0.008
HOMA-IR	5.4 ± 4.8	4.8 ± 4.1	5.5 ± 6.0	5.3 ± 3.6	6.4 ± 5.2	0.347
Muscle mass (cm^2^)	121.0 ± 32.5	115.4 ± 32.5	114.3 ± 23.7	122.8 ± 29.4	141.2 ± 38.0	0.007
Visceral fat (cm^2^)	119.2 ± 69.8	81.3 ± 54.6	130.7 ± 70.4	137.8 ± 52.2	168.7 ± 69.4	<0.001

Data were reported as mean ± standard deviation or median [interquartile range] for continuous variables and proportion (%) for categorical variables. PT-INR, prothrombin time-international normalized ratio; HbA1c, glycated hemoglobin; FIB-4, Fibrosis-4; APRI, aspartate transaminase to Platelet Ratio Index; NAFLD, nonalcoholic fatty liver disease; HOMA-IR, Homeostatic Model Assessment of Insulin Resistance.

**Table 3:** Linear regression analysis for steatosis of CAP score.

Variable	Univariable		Multivariable	
	B (95% CI)	*p*	B (95% CI)	*p*
Age	−1.393 (−1.928, −0.858)	<0.001	0.124 (−0.343, 0.593)	0.599
Sex				
Male	0			
Female	−23.360 (−38.066, −8.654)	0.002	2.400 (−16.158, 20.959)	0.798
Height (cm)	1.772 (0.960, 2.584)	<0.001		
Weight (kg)	1.969 (1.616, 2.322)	<0.001		
Etiology				
Viral	0			
Non-viral	30.227 (4.612, 55.842)	0.020	−15.786 (−34.869, 3.296)	0.104
Etiology				
Hepatitis B virus	0			
Hepatitis C virus	56.116 (−14.891, 127.124)	0.120		
Alcohol	12.164 (−18.994, 43.322)	0.442		
Etc.	43.341 (16.338, 70.344)	0.002		
White blood cell (/μL)	0.002 (0, 0.005)	0.079		
C-reactive protein (mg/dL)	−5.965 (−9.573, −2.357)	0.001	−2.883 (−5.402, −0.364)	0.025
Hemoglobin (g/dL)	12.208 (9.014, 15.401)	<0.001	−2.024 (−0.621,2.162)	0.341
Platelet (1 k/μL)	0.225 (0.134, 0.317)	<0.001	0.012 (−0.070, 0.095)	0.760
Aspartate aminotransferase (U/L)	−0.001 (−0.012, 0.010)	0.824		
Alanine aminotransferase (U/L)	−0.006 (−0.021, 0.008)	0.421		
Total bilirubin (mg/dL)	−3.762 (−7.506, −0.018)	0.048	−1.833 (−5.403, 1.767)	0.312
Albumin (g/dL)	51.239 (40.475, 62.004)	<0.001	13.908 (0.767, 27.048)	0.038
Serum sodium (mmol/L)	5.176 (2.801, 7.550)	<0.001	0.015 (−1.797, 1.827)	0.986
Creatinine (mg/dL)	−3.120 (−20.843, 16.602)	0.729		
Total cholesterol (mg/dL)	0.375 (0.233, 0.516)	<0.001	0.090 (−0.016, 0.197)	0.095
High density lipoprotein (mg/dL)	0.087 (−0.339, 0.514)	0.686		
Low density lipoprotein (mg/dL)	0.497 (0.309, 0.684)	<0.001		
Triglyceride (mg/dL)	0.092 (0.045, 0.138)	<0.001	0.027 (−0.005, 0.060)	0.100
PT-INR	−13.418 (−35.093, 8.256)	0.223		
Glucose (mg/dL)	−0.076 (−0.213, 0.060)	0.270		
Diabetes	15.211 (−1.667, 32.091)	0.077		
HbA1c (%)	−14.893 (−26.908, −2.878)	0.015		
Insulin (uIU/mL)	0.817 (0.303, 1.330)	0.001		
Fibroscan^®^ stiffness	−0.969 (−1.158, −0.359)	0.002	0.027 (−0.596, 0.651)	0.930
Fatty liver grade				
Normal	0			
Mild	37.883 (24.383, 51.383)	<0.001	31.756 (17.551, 48.962)	<0.001
Moderate	90.680 (76.654, 104.707)	<0.001	64.809 (47.896, 81.721)	<0.001
Severe	110.359 (96.017, 124.701)	<0.001	88.468 (70.557, 106.380)	<0.001
Skeletal muscle mass (kg)	3.807 (2.719, 4.894)	<0.001		
Skeletal muscle mass (%)	−4.572 (−6.044, −3.100)	<0.001	−4.261 (−8.696, 0.172)	0.059
Muscle mass (cm^2^)	0.462 (0.146, 0.777)	0.004		
Body fat mass (kg)	3.249 (2.668, 3.830)	<0.001		
Body fat mass (%)	3.169 (2.404, 3.935)	<0.001		
Visceral fat (cm^2^)	0.390 (0.253, 0.527)	<0.001		
Fat mass index (kg/m^2^)	8.267 (6.581, 9.953)	<0.001	−2.181 (−10.295, 5.391)	0.596
Total body water (L)	3.088 (2.178, 3.998)	<0.001	−18.295 (−57.785, 21.194)	0.361
Base metabolic rate (kcal)	0.105 (0.074, 0.136)	<0.001	0.695 (−0.626, 2.017)	0.300
Waist to hip ratio	406.962 (321.548, 492.375)	<0.001	1.800 (−135.699, 139.299)	0.979
Body mass index	6.472 (5.268, 7.676)	<0.001	−0.164 (−4.270, 3.941)	0.937
FIB-4 score	−0.931 (−1.902, 0.039)	0.060		
APRI score	−0.108 (−1.065, 0.849)	0.824		
NAFLD fibrosis score	−5.826 (−9.071, −2.582)	<0.001		
HOMA-IR	2.142 (0.550, 3.734)	<0.001	0.632 (−0.417, 1.682)	0.236

PT-INR, prothrombin time-international normalized ratio; HbA1c, glycated hemoglobin; FIB-4, Fibrosis-4; APRI, aspartate transaminase to Platelet Ratio Index; NAFLD, nonalcoholic fatty liver disease; HOMA-IR, Homeostatic Model Assessment of Insulin Resistance; B, regression coefficient; CI, confidence interval.

## Data Availability

The datasets generated during and/or analysed during the current study are available from the corresponding author on reasonable request.

## Supplementary Materials

The following supporting information can be downloaded at: https://www.mdpi.com/article/10.3390/healthcare10112268/s1, Figure S1: Factors associated with CAP score (multivariate analysis).
