# Supplementary figures and images for: Bioelectrical Impedance Analysis Can Be an Effective Tool for Screening Fatty Liver in Patients with Suspected Liver Disease
Source: Healthcare (Basel). 2022 Nov 11;10(11):2268. doi: 10.3390/healthcare10112268 (PMC9690130; doi:10.3390/healthcare10112268)

**Supplementary Figure S1.** Factors associated with CAP score (multivariate analysis)

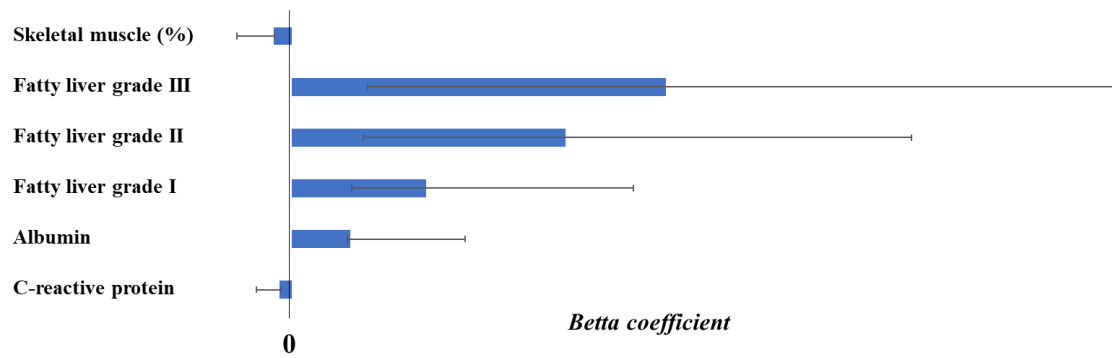

Supplement: Supplementary file 1 [file healthcare-10-02268-s001.zip › healthcare-1990683-supplementary.pdf]
